# Identification and validation of ferroptosis-related lncRNA signature as a prognostic model for skin cutaneous melanoma

**DOI:** 10.3389/fimmu.2022.985051

**Published:** 2022-09-29

**Authors:** Sen Guo, Jianru Chen, Xiuli Yi, Zifan Lu, Weinan Guo

**Affiliations:** ^1^ Department of Dermatology, Xijing Hospital, Fourth Military Medical University, Xi’an, China; ^2^ Department of Biopharmaceuticals, School of Pharmacy, Fourth Military Medical University, Xi’an, China

**Keywords:** cutaneous melanoma, immune microenvironment, immunotherapy, ferroptosis, lncRNA

## Abstract

**Background:**

Melanoma is a type of skin cancer, which originates from the malignant transformation of epidermal melanocytes, with extremely high lethality. Ferroptosis has been documented to be highly related to cancer pathogenesis and the effect of immunotherapy. In addition, the dysregulation of lncRNAs is greatly implicated in melanoma progression and ferroptosis regulation. However, the significance of ferroptosis-related lncRNA in melanoma treatment and the prognosis of melanoma patients remains elusive.

**Methods:**

*Via* Least Absolute Shrinkage Selection Operator (LASSO) regression analysis in the TCGA SKCM database, a cutaneous melanoma risk model was established based on differentially-expressed ferroptosis-related lncRNAs (DEfrlncRNAs). The nomogram, receiver operating characteristic (ROC) curves, and calibration plots were conducted to examine the predictive performance of this model. Sequentially, we continued to analyze the differences between the high- and low-risk groups, in terms of clinical characteristics, immune cell infiltration, immune-related functions, and chemotherapy drug sensitivity. Moreover, the expressions of DEfrlncRNAs, PD-L1, and CD8 were also examined by qRT-PCR and immunohistochemical staining in melanoma tissues to further confirm the potential clinical implication of DEfrlncRNAs in melanoma immunotherapy.

**Results:**

16 DEfrlncRNAs were identified, and a representative risk score for patient survival was constructed based on these 16 genes. The risk score was found to be an independent prognostic factor for the survival of melanoma patients. In addition, the low-risk group of patients had higher immune cell infiltration in the melanoma lesions, higher sensitivity to chemotherapeutic agents, and a better survival prognosis. Besides, the high expression of the identified 5 DEfrlncRNA in the low-risk group might suggest a higher possibility to benefit from immune checkpoint blockade therapy in the treatment of melanoma.

**Conclusion:**

The DEfrlncRNA risk prediction model related to ferroptosis genes can independently predict the prognosis of patients with melanoma and provide a basis for evaluating the response of clinical treatment in melanoma.

## Introduction

Melanoma is a type of skin cancer, which originates from the malignant transformation of epidermal melanocytes, with high lethality. Melanoma though accounts for only approximately 4% incidences of all skin malignancies, it results in 80% of the mortalities (1, 2), as its high invasiveness frequently contributes to the metastases in the brain, liver, lung, and other vital organs (3–6). While early-stage melanomas are curable through surgical resection, metastatic melanomas that have spread to multiple organs are extremely challenging to treat (7). The prognosis of melanoma patients has been substantially improved with the revolutionary progress in developing targeted therapy and immunotherapy. However, that tumor cell heterogeneity-induced challenges in identifying the sensitive subpopulations, low response rates, and treatment resistance have significantly hampered the efficacy of currently available therapies (8–10). Thus, it is important to explore and develop alternative potential biomarkers to provide a more accurate diagnosis and prognosis prediction and more individualized treatment of melanoma.

Long non-coding RNAs (lncRNAs) have been identified as RNAs incorporating more than 200 nucleotides and are highly involved in regulating the expression levels of downstream genes (11). Aberrantly expressed lncRNAs participate in manipulating cell biology and disease processes through epigenetics (chromosome silencing, genomic imprinting, and chromatin modification), signaling pathway transduction, and alternative non-coding RNAs such as microRNAs (miRNAs) that are involved in modulating gene expression (12). Previous investigations revealed that lncRNAs are closely related to the initiation and progression of nearly all human cancers, including melanoma. In addition, dysregulated lncRNAs play versatile roles in different aspects of tumor cell biological activities, ranging from tumor cell proliferation, invasion, and metastasis to angiogenesis (13–15). Accordingly, lncRNAs might be promising targets for melanoma treatment.

Ferroptosis is a recently-identified form of regulated cell death. The characteristics of ferroptosis include iron-dependent peroxidation and the accumulation of various reactive compounds such as oxidized polyunsaturated fatty acids (PUFAs), lipid peroxides, and reactive oxygen species (ROS) (16). Recently, ferroptosis is reported to be closely associated with regulating tumor growth and the treatment outcome of radiotherapy, chemotherapy, and immunotherapy (17). Therefore, ferroptosis-triggering drugs might robustly improve the anti-tumor efficacy of these therapies. Specifically, Tsoi *et al.* has shown that erastin, a ferroptosis inducer, could significantly potentiate melanoma cell death caused by BRAF inhibitors (18). In addition, it was reported that miR-324-3p reversed cisplatin resistance through the induction of ferroptosis in a GPX4-dependent manner in lung adenocarcinoma (19). Furthermore, the inhibition of GP4 might induce ferroptosis, which enhanced triple-negative breast cancer’s sensitivity to the drug gefitinib (20).

More importantly, ferroptosis also has a critical role in anti-tumor immunity and the tumor microenvironment (21). On one hand, the induction of ferroptosis leads to the exposure of tumor antigens, which enhances tumor cell immunogenicity and increases immunotherapy efficacy (22). On the other hand, interferon-γ from CD8^+^T cells induces lipid peroxides and consequently ferroptosis in cancer cells by suppressing the expression of systemic Xc^-^ (cysteine/glutamate antiporter) (23, 24). These reports indicated that precise induction of ferroptosis is an encouraging combinatorial strategy that might be useful in cancer-targeted therapy and immunotherapy. Intriguingly, a recent report supported lncRNAs as a critical regulatory paradigm in ferroptosis and an important factor associated with the therapeutic outcomes of cancer (25). However, the involvement of ferroptosis-associated lncRNAs in the progression of melanoma is still elusive. Therefore, the significance of ferroptosis-related lncRNAs concerning the treatment of melanoma patients and their prognosis requires additional investigation.

This investigation explored ferroptosis-related lncRNAs and evaluated potential prognostic biomarkers in melanoma using bioinformatics methods. We employed The Cancer Genome Atlas (TCGA) skin cutaneous melanoma (SKCM) and Genotype-Tissue Expression (GTEx) databases to evaluate differentially-expressed lncRNAs associated with ferroptosis in melanoma. Sixteen lncRNAs were identified to be closely related to the prognosis of melanoma patients. Subsequently, a melanoma risk prediction model was developed, and its application in predicting patients’ diagnosis, prognosis, chemotherapy response, and tumor immune evasion was evaluated.

## Materials and methods

### Retrieval of transcriptome data, its processing, and analysis of differential gene expression

In this study, the data obtained from transcriptome profiling (RNA-Seq) was paired with fragments per kilobase million (FPKM) obtained from SKCM patients and normal controls that were primarily obtained from TCGA (https://portal.gdc.cancer.gov/) as well as the Genotype-Tissue Expression (GTEx) database (https://toil.xenahubs.net/download/GTEX_phenotype.gz). The Gene transfer format (GTF) files were acquired from Ensembl (http://asia.ensembl.org) for annotation, in order to identify the lncRNAs and mRNAs for further assessment. The R package “edgeR” was utilized to screen for differentially-expressed lncRNAs (DElncRNAs) in melanoma samples as well as samples obtained from normal tissues (26). Sixty-three genes closely related to ferroptosis were downloaded (27) and used to identify ferroptosis-related lncRNAs (frlncRNAs) through a co-expression strategy. The R package “psych” was employed to analyze the relationship between the lncRNAs and the ferroptosis-related genes (28). Any ferroptosis gene correlation coefficients that were > 0.6 and exhibited a *P*-value < 0.05 were designated as frlncRNAs. For the identification of the DEfrlncRNAs, we intersected the positively correlated frlncRNAs and the up-regulated DElncRNAs to obtain the DEfrlncRNAs that were candidate genes for subsequent analysis.

### Patient clinical data acquisition

Clinical and prognostic data of melanoma patients were accessed from the SKCM project associated with TCGA. The information from 458 patients with known survival times was obtained.

### Establishment of a prognostic risk score model using DEfrlncRNAs

Univariate Cox analysis was utilized in the identification of possible relationships between the DEfrlncRNA profiles and patient overall survival (OS). This was accomplished through the utilization of the “survival” package and a *P*-value < 0.05 (29). The prognosis-related DEfrlncRNAs were determined by conducting the least absolute shrinkage and selection operator (LASSO) regression, and the “glmnet” R package was used for the prevention of overfitting (30). Subsequently, multivariate Cox analysis was utilized to assess the DEfrlncRNAs found to be related to prognosis to develop a prognostic DEfrlncRNA signature and to calculate the coefficients. A forest map was created to visualize the data obtained with the multivariate Cox regression analysis. The formula described below was utilized to establish a prognostic risk model:


$$Risk\;score=\sum_{i=1}^{n}\left(Coef_{i}\times x_{i}\right)$$

Concerning the variables associated with the formula, Coef represents the multivariate Cox regression analysis coefficient of DEfrlncRNAs, while X indicates the relative expression levels of DEfrlncRNA. The patients were categorized into a high-risk (HR) or a low-risk (LR) group, with the median RS serving as the division between the two groups.

### Risk model validation

The model’s predictive value was evaluated with Kaplan-Meier method to assess the differences in survival between the HR and LR groups. In addition, univariate and multivariate Cox regression analyses were carried out to determine if the model was an independent factor in the survival of SKCM patients. To confirm the applicability of the model for use in clinical practice, we used the chi-square test to evaluate if there was any significant relationship between clinicopathological characteristics and the model. The differences in RS that occurred among the groups that presented different clinicopathological characteristics were compared using the Wilcoxon signed-rank test. Box and scatter diagrams were prepared to demonstrate the results of the analysis. Furthermore, the RS was combined with the clinicopathological features of the T and N stages to develop a nomogram that could estimate the 2-, 4-, and 5-year survival of SKCM patients. A calibration curve was utilized to determine if the rate of survival that was predicted was actually in good agreement with the reported rate of patient survival.

### Immune cell intratumoral infiltration estimation

To analyze the association that was present between the infiltrating immune cells and the SKCM RS, the CIBERSORT package was utilized to determine the level of immune cell infiltration in the included SKCM samples (31). A permutation number of 1,000 was used. Samples that exhibited a *P*-value < 0.05 in the results obtained from the CIBERSORT analysis were utilized in additional analyses. In addition, to examine the model’s clinical performance further, we examined the relationships between the expression levels of the immune checkpoints and this model.

### Clinical performance of treatment

To evaluate the usefulness of this model for melanoma treatment in clinical practice, we determined the IC50 for 94 SKCM-related drugs according to the Cancer Genome Project (CGP) website data with the “pRRophetic” R package (32). Any differences observed between the HR and LR groups in the IC50s were evaluated using the Wilcoxon signed-rank test, with the subsequent results displayed using box drawings.

### Clinical specimens

Tissue samples that were utilized for qRT-PCR assays and immunohistochemical staining were collected from 30 melanoma patients and 20 cases of nevus that were confirmed histologically. The clinical specimens were provided by the Department of Dermatology, Xijing Hospital, Fourth Military Medical University. The protocol used for this study was developed and performed in compliance with the principles of the Declaration of Helsinki. Furthermore, this study was approved by the Ethics Review Board of Fourth Military Medical University. All patients provided written informed consent.

### RNA extraction and qRT-PCR

TRIzol reagent (cat. 15596018, Invitrogen, CA, USA) was used to extract total RNA. mRNA was reverse transcribed to cDNA using the PrimeScriptTM RT Master Mix kit (cat. RR036A, TaKaRa, Japan). qRT-PCR was performed using a SYBR Premix Ex TaqTM II kit (cat. RR820A, TaKaRa, Tokyo, Japan). qRT-PCR analysis was conducted on a BIO-RAD Multicolor Real-time PCR Detection System (iQTM5, Bio-Rad, CA, USA). The primer pairs were: LINC01281 forward, 5′- TTAAGGCAGCGAGAAGTGGT -3′ and LINC01281 reverse, 5′- TGGCACTTGAACCTCACAACA -3′; FAM30A forward, 5′- CGTGTTGAGCTTTGCACCCT-3′ and FAM30A reverse, 5′- TGTGGCTCTTCATTCACCCT-3′; LINC00861 forward, 5′- GGACCGATAGGGCGATTAAACT -3′ and LINC00861 reverse, 5′- CCTCCTGGACTCGTGTAAGA -3′; LINC01727 forward, 5′- TACAGAACTGGTTGCTGCCTC -3′ and LINC01727 reverse, 5′- AGCATCCCAGTGAGGTCTGAA -3′; PIK3D-AS1 forward, 5′- CAGCCCACTCCAGTGTCTTC -3′ and PIK3CD-AS1 reverse, 5′- TGGCCTGCTGGAGTTTCATT -3′; β-actin forward, 5′- TCATGAAGTGTGACGTGGACATC -3′ and β-actin reverse, 5′- CAGGAGGAGCAATGATCTTGATCT -3′. Relative quantification was carried out using the ΔΔCT method. The data were presented in the linear form through the use of the formula 2^-ΔΔCT^. β-actin mRNA served as the internal control.

### Immunohistochemical staining analysis

CD8α and PD-L1 expression was measured in melanoma patient tissue samples that were embedded in paraffin. The paraffin-embedded tissues were sectioned on a microtome and mounted on glass microscope slides. The sections were de-paraffinized and then rehydrated through a graded series of ethanol solutions. Antigen retrieval was carried out using Tris-EDTA buffer (0.05% Tween 20, 1 mM EDTA Solution, 10 mM Tris Base, pH 9.0), and then goat serum was applied for 30 mins to inhibit non-specific binding. The tissue sections were incubated in a solution containing antibodies to CD8 (cat. ZA-0508, rabbit monoclonal antibody, 1:1, ZSGB-BIO, China) or antibodies to PD-L1 (cat. ab228415, rabbit monoclonal anti-PD-L1 antibody, 1:200, Abcam, Cambridge, MA, USA) overnight at 4°C. Subsequently, the tissue sections were exposed to an anti-rabbit alkaline phosphatase secondary antibody (cat. cw2069s, 1:1, Cwbio, China). Finally, the sections were exposed to Fast Red solution, counterstained using hematoxylin, and glycerol-mounted coverslips were applied. The protocol used to evaluate the staining has been described previously (33). The proportions of positive-stained cells were determined and then subdivided into 4 grades: 3 (67-100%), 2 (34-66%), 1 (1-33%) and 0 (0%). The staining intensities also were sub-divided into 4 different categories or groups: 3 (strong), 2 (moderate), 1 (weak), and 0 (none). The final scores used to express the level of staining were determined to be the product of the score for the percentage of positively staining cells and the score for the level of staining intensity.

### Statistical analysis

Each experiment was carried out a minimum of three times. The data were analyzed statistically using unpaired, two-tailed Student’s *t*-tests using GraphPad Prism v3.0. One-way ANOVA analysis was utilized to assess any differences that occurred among the multiple groups. Pearson correlation was utilized to determine the presence of any significant associations that occurred between the expression levels of two genes. All data were presented as mean ± S.D. *P* value < 0.05 was deemed to be significant.

## Results

### Identification of differentially-expressed ferroptosis-associated lncRNAs

**Figure 1:**
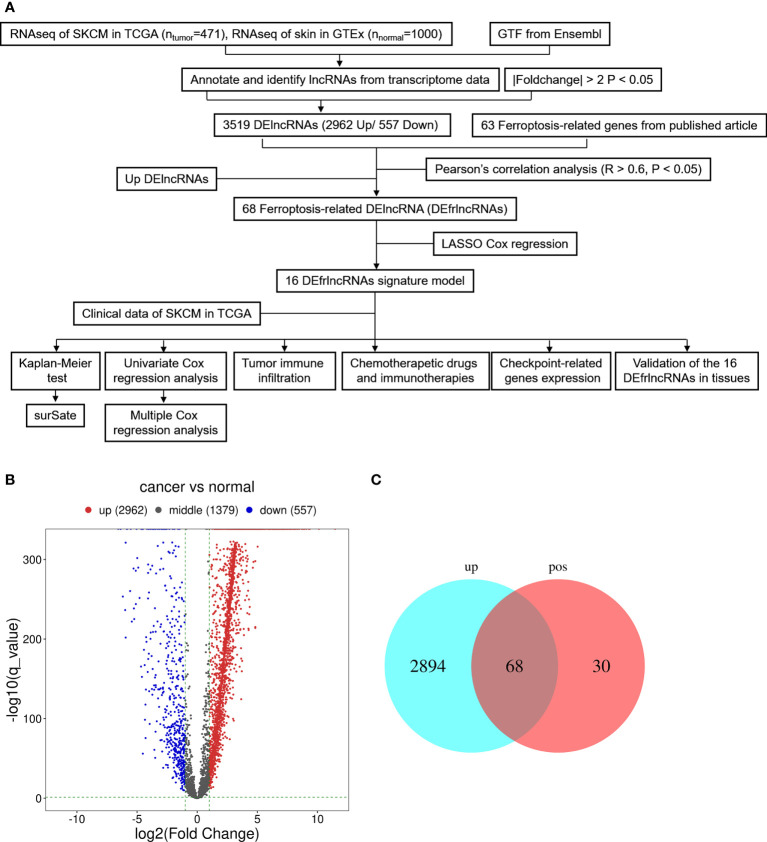
Presentation of differentially expressed lncRNAs between SKCM samples and samples from normal tissues (log2 fold change>2, adjusted p-value<0.05). **(A)** Flow chart of the analytical process in this study. **(B)** A volcano map of lncRNAs that are differentially expressed. Red abd blue dots represent the genes that are significantly up-regulated and downregulated, respectively. **(C)** A Venn map for the up-regulated ferroptosis-related lncRNAs.

The graphic flow chart briefly displayed the design of the present study in **Figure 1A**. We retrieved the transcriptome profiles for melanoma and normal controls from the skin cutaneous melanoma (SKCM) project of the TCGA database as well as the GTEx database. Four hundred and seventy-one tumors and 1,000 normal samples were ultimately included in this study. The data were annotated based on the GTF files obtained from Ensembl, which were utilized to determine the presence of differential expression. Subsequently, based on the set cutoff criteria of |fold-change| > 2 and *P* < 0.05, 4,898 lncRNAs were assessed and 3,519 lncRNAs exhibited differential expression when melanoma tissues were compared to normal tissues (2,962 up-regulated and 557 down-regulated). A volcano map was used to visualize the expression profiles of the DElncRNAs (**Figure 1B**). Then, the co-expression analysis was performed to compare the known ferroptosis-related genes and the DElncRNAs. Ninety-eight ferroptosis-associated lncRNAs were determined to be positively correlated with canonical ferroptosis-associated genes (**Table S1**), of which the expression of 68 lncRNAs was significantly up-regulated in melanoma samples compared to the controls (**Figure 1C**), and these lncRNAs were designated as up-regulated DEfrlncRNAs.

### Construction of the DEfrlncRNA predictive signature

**Figure 2:**
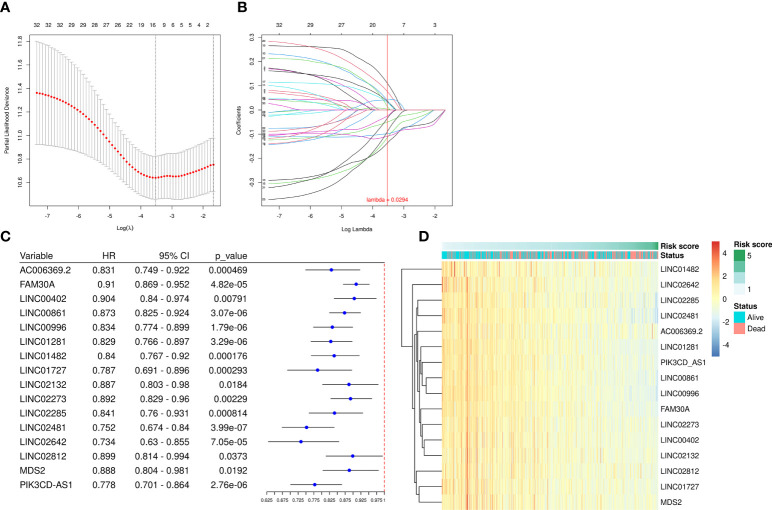
A prognostic risk model was established using LASSO regression and Cox regression analyses. **(A)** Cross-validation was used to tune the parameter screening in the LASSO regression model. **(B)** The LASSO coefficient profiles for the 32 DEfrLncRNAs. **(C)** Forest plots of HRs and *P*-values of selected DEfrLncRNAs using univariate Cox regression analysis. Sixteen of the DEfrLncRNAs were found to be prognostic factors, and all are protective factors in SKCM with HR< 1. **(D)** Distribution heat map of the 16 DEfrLncRNA expressions, RS, and clinical status (alive or dead) relative to SKCM.

We employed the univariate Cox regression method to analyze the 68 up-regulated DEfrlncRNAs, and 32 of them were identified at *P* < 0.05. A prognostic classifier was established using the LASSO Cox regression model. A vertical dotted line was placed to indicate the value chosen through 10-fold cross-validation (**Figures 2A, B**). The optimal λ value, which was 0.0294, with log(λ) = -3.53 resulted in 16 non-zero coefficients (**Figure 2C**). The expression levels of 16 DEfrlncRNAs in the TCGA SKCM database were displayed in **Figure 2D**. The RS was determined as follows: RS = (-0.025 × LINC00861 expression) + (-0.034 × PIK3CD-AS1 expression) + (-0.050 × FAM30A expression) + (0.089 × LINC02642 expression) + (-0.098 × LINC01482 expression) + (-0.108 × LINC02481 expression) + (-0.113 × LINC01281 expression) + (-0.136 × LINC00996 expression) + (0.110 × LINC02132 expression) + (0.076 × LINC02273 expression) + (0.041 × MDS2 expression) + (0.039 × LINC00402 expression) + (0.035 × AC006369.2 expression) + (0.030 × LINC01727 expression) + (0.013 × LINC02285 expression) + (0.003 × LINC02812 expression). Besides, we employed Pearson correlation analysis to validate the relationship between 16 DEfrlncRNAs and ferroptosis-related genes, which displayed that the expressions of DEfrlncRNAs were in negative correlation with most of the ferroptosis-associated molecules (**Supplementary Figure 1**).

### Predictive signature and SKCM patient prognosis correlations

**Figure 3:**
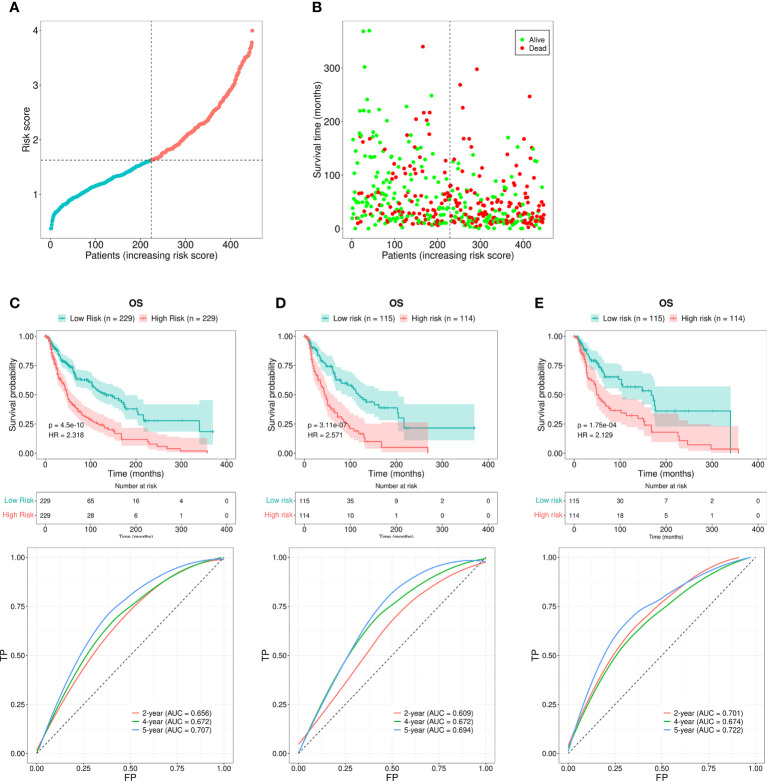
Risk assessment model for prognosis prediction. **(A)** The RS distribution for the SKCM patients. **(B)** The number of patients with different RS who were alive or had died. A larger number of deaths were observed in the group with a higher RS. **(C)** The OS rates in patients with SKCM who were included in the LR and HR groups were evaluated using the Kaplan-Meier method. The ROC curves as well as the AUCs at 2-, 4-, and 5-year survival as the predictive signature. **(D)** The OS rate for SKCM patients in the first internal cohort, as assessed using the Kaplan-Meier method; ROC curves and AUCs at 2-, 4-, and 5-year survival as the predictive signature. **(E)** The SKCM patient OS rate in the second internal cohort was determined using the Kaplan-Meier method; the ROC curves and AUCs at 2-, 4-, and 5-year survival as the predictive signature.

Each patient’s RS was calculated using the formula mentioned above. The patients were subdivided into the HR or the LR group according to the median RS value. The RS values of the HR and LR group were shown in **Figure 3A**. Higher RS values indicated higher mortality (**Figure 3B**). The assessment based on the Kaplan-Meier curve revealed that LR patients possessed better OS than HR patients (*P* < 0.001; **Figure 3C**). The AUCs for 2-, 4-, and 5-year survival rates were 0.656, 0.672, and 0.707, respectively, which was indicative of acceptable predictive performance (**Figure 3C**).

**Figure 4:**
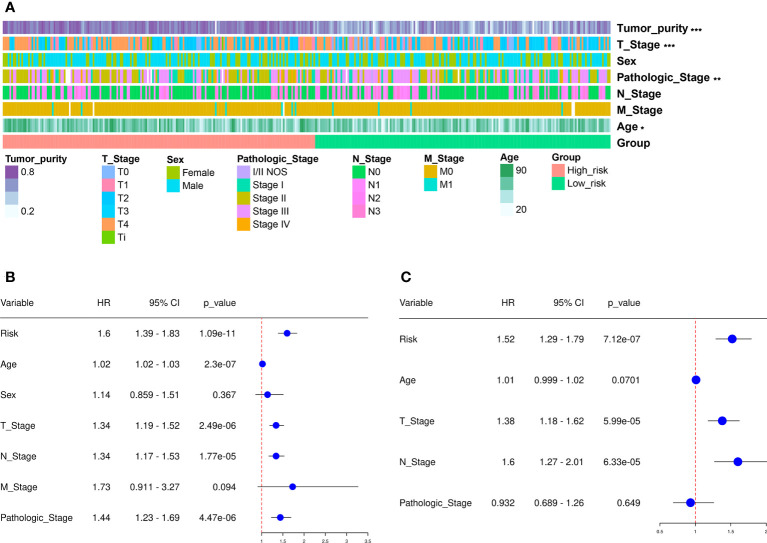
Clinical assessment using the risk assessment model. **(A)** A heatmap reveals the distribution of tumor purity, T stage, sex, pathologic stage, N and M stages, and age, along with RS. **(B)** The univariate Cox regression analysis was used to examine the associations among the RS, clinical features, and OS of SKCM patients. **(C)** Multivariate Cox regression analysis was employed to reveal the associations present among the RS, clinical features, and OS of SKCM patients. *p < 0.05, **p < 0.01, ***p < 0.001.

We next divided the 458 SKCM patients randomly into two groups (n = 229 per group) and verified the predictive signature for OS in these two groups. The OS rate observed in the HR group was lower than the LR group for the first internal cohort, which was consistent with the results observed in the entire dataset (**Figure 3D**). Concerning the second internal cohort, the prognosis observed in the HR group was worse compared to that in the LR group (**Figure 3E**). The ROC curves obtained from the two cohorts revealed a reasonable predictive performance. Concerning the first internal cohort, the AUCs associated with the 2-, 4-, and 5-year survival were 0.701, 0.674, and 0.722, respectively (**Figure 3D**). The AUCs of the second internal cohort concerning the 2-, 4-, and 5-year survival were 0.609, 0.672, and 0.694, respectively (**Figure 3E**). Furthermore, chi-square tests were employed to determine the associations between the clinicopathological characteristics and the prognosis of SKCM patients. The clinicopathological characteristics include age, gender, pathologic stage, T/M/N stages, and tumor purity. As revealed by the strip chart and the subsequently constructed scatter diagrams that were generated using the Wilcoxon signed-rank test, the T stage, pathologic stage, age, and tumor purity were markedly associated with the RS (**Figure 4A**).

Cox regression analysis was carried out to assess if the predictive signature was an independent prognostic factor for SKCM patients. The univariate Cox assessment revealed that the RS (*P* < 0.001), N stage (*P* < 0.001), age (*P* < 0.001), T stage (*P* < 0.001), and pathologic stage (*P* < 0.001) correlated significantly with OS (**Figure 4B**). Multivariate Cox analysis further suggested that T stage ( *P* < 0.001), N stage (*P* < 0.001), and RS (*P* < 0.001) were independent prognostic predictors for SKCM patients (**Figure 4C**).

**Figure 5:**
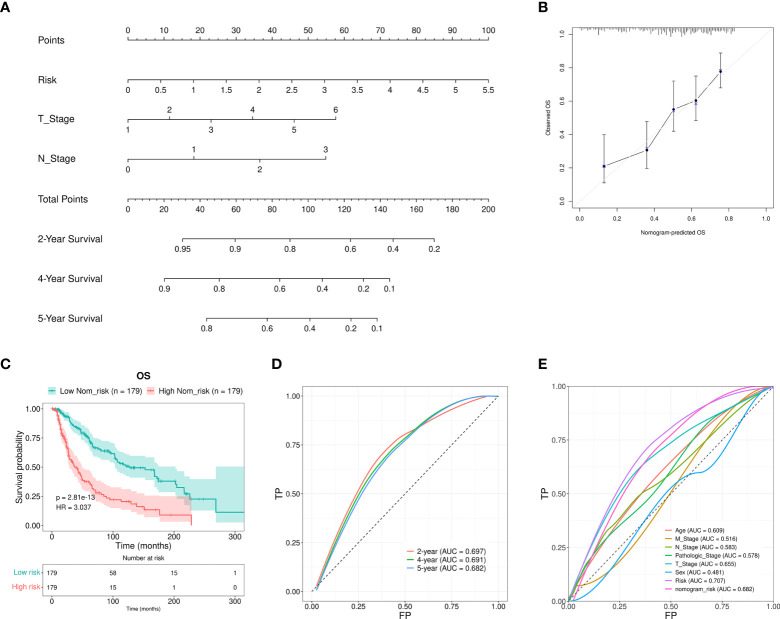
Nomogram construction and verification. **(A)** A nomogram that combined the clinical parameters and RS was applied to estimate the 2-, 4-, and 5-year OS for patients with SKCM. **(B)** The consistency of the calibration curve tests between the observed rates of OS and the survival rates that were predicted. **(C)** The OS rates for the nomogram samples for the low and high Nom-risk groups were analyzed using the Kaplan-Meier method. **(D)** The ROC curves and the AUCs at 2-, 4-, and 5-year survival as the predictive signature. **(E)** ROC curve of the RS and clinicopathological features.

Furthermore, a nomogram that included the T stage, N stage, and the RS was developed to predict the prognosis of SKCM patients. These values were *P <* 0.01 in the multivariate Cox regression analysis. The constructed nomogram was able to predict the 2-, 4-, and 5-year prognosis for SKCM patients (**Figure 5A**). The calibration curves revealed excellent consistency between the predicted survival rates and the observed OS rates (**Figure 5B**). The OS of the HR group was significantly reduced in comparison to the LR group (**Figure 5C**). The AUCs for the 2-, 4-, and 5-year survival were 0.697, 0.691, and 0.682, respectively. These results suggested a reasonable predictive performance (**Figure 5D**). The RS AUC was 0.707. Thus, compared with other clinicopathological variables, nomogram RS exhibited stronger potential in predicting the prognosis of SKCM patients. (**Figure 5E**).

### Immune cell infiltration and immune-related signature differences in the SKCM database between the LR and HR groups

**Figure 6:**
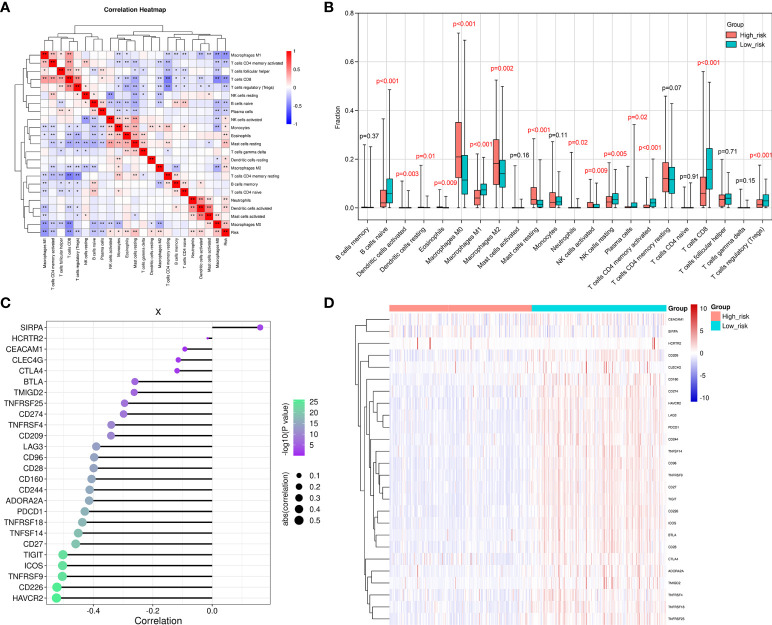
The difference observed between the HR and LR groups in the immune microenvironment. **(A)** A correlation heatmap for the 22 different types of immune cells. The degree of correlation is represented by the size of the colored squares. Blue indicates the existence of a negative correlation, while red indicates a positive correlation. The intensity of the color represents the strength of the correlation; a darker color represents a stronger correlation. **(B)** The CIBERSORT algorithm was employed to determine the level of infiltration that the 22 immune cells exhibited in the LR and HR groups. **(C)** This figure reveals the correlation between the RS and the immune checkpoint genes. The dot size indicates how strong the correlation is between the RS and the immune checkpoint genes. Larger dots represent stronger correlations, and smaller dots indicate weaker correlations. Furthermore, the dot color and its intensity is indicative of the *P*-value. A more intense purple color represents a lower *P*-value, and a more intense green color represents a larger *P*-value. A *P*-value<0.05 indicates statistical significance. **(D)** A distribution heat map that shows the expression of immune checkpoint genes in the LR and HR groups. *p < 0.05, **p < 0.01.

The correlations between 22 different types of infiltrating immune cells and the observed RS were analyzed using Pearson correlation analysis to determine to which extent the immune components were affected by the RS. The RS was observed to positively correlate with the number of infiltrated M0 and M2 macrophage, resting mast cell, activated dendritic cell, resting dendritic cell, eosinophil, neutrophil, activated NK cell, and monocyte levels. Furthermore, they were negatively correlated with the levels of CD8^+^T cells, M1 macrophages, naive B cells, activated memory CD4^+^T cells, resting NK cells, and regulatory T cells (Tregs) (**Figure 6A**). We also assessed the infiltration degree of 22 different types of immune cells in the HR and LR groups (**Figure 6B**). The numbers of resting mast cells, M2 and M0 macrophages, eosinophils, neutrophils, resting and activated dendritic cells, activated NK cells, and monocytes were increased in the HR group. In addition, the activated CD4^+^ memory T cells, CD8^+^T cells, M1 macrophages, naïve B cells, regulatory T cells (Tregs), and resting NK cells exhibited decreases in the HR group. These results suggested that the SKCM RS was related to immune cell infiltration in the microenvironment.

We went on to explore the relationship between the levels of multiple immune checkpoint molecules and the SKCM RS (**Figures 6C, D**). It was noted that PD-L1 (*CD274*) was up-regulated in the SKCM LR group. *CD274* is a classic immune checkpoint gene that exhibits constitutive expression in tumor cells and can be targeted by clinically approved drugs (34). In contrast, an increased number of infiltrating CD8^+^ T cells was observed when the SKCM RS decreased. This result indicated that samples from SKCM patients in the LR group presented a relative abundance of CD8^+^T cells. However, inhibitory receptor overexpression, including PD-L1, constrained the cytotoxic function of CD8^+^ T cells. Therefore, PD-L1 inhibitors might be potential therapeutic strategies in these patients. We also evaluated the immune-related signature activities between HR and LR groups by gene set variation analysis (GSVA), which displayed that patients in HR group exhibited suppressed natural killer cell-mediated cytotoxicity and B cell receptor signaling pathway activities (**Supplementary Figure 2**).

### The sensitivity to common chemotherapeutic drugs for patients exhibiting different SKCM RS

We also investigated the efficacy of chemotherapy in patients of different RS groups, which was exhibited by a range of common chemotherapeutic drugs used to treat melanoma in the SKCM dataset of the TCGA project. Nilotinib, methotrexate, rapamycin, and cisplatin exhibited higher IC50s in the HR group (**Supplementary Figures 3A–D**). These findings demonstrate that the model might predict the tumor chemosensitivity of patients with melanoma.

### Validation of the lncRNAs in melanoma tissues

**Figure 7:**
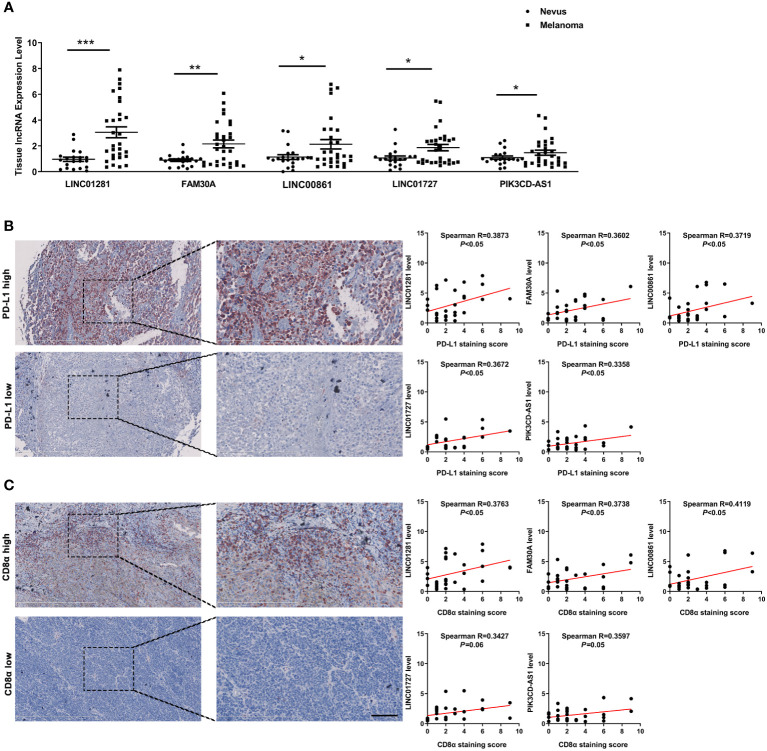
**(A)** The expression of five DEfrlncRNAs in melanoma tissues and nevi. **(B, C)** Correlation analysis between the five DEfrlncRNAs and PD-L1/CD8α in melanoma tissues. The Spearman correlation was used to calculate the *r* value. *p < 0.05, **p < 0.01, ***p < 0.001.

To further confirm the potential clinical implications of DEfrlncRNAs in melanoma immunotherapy, qRT-PCR analysis was conducted to determine the expression levels of the top five highly-expressed DEfrlncRNAs in a cohort of 30 melanoma tissues. The levels of the five DEfrlncRNAs were prominently increased in tumors compared with nevi (**Figure 7A**). We further assessed the expression of PD-L1 and CD8 by immunohistochemistry in these melanoma tissues. As a result, a positive correlation between the levels of the five DEfrlncRNAs and the scores for immunohistochemical staining for PD-L1 and CD8 was observed (**Figures 7B, C**). These results supported the close association between DEfrlncRNAs and CD8^+^T cell-dependent anti-tumor immunity.

## Discussion

Numerous promising drugs that target specific molecules have emerged in the treatment of SKCM, like inhibitors of BRAF, MEK, CTLA-4, and PD-1 (35). However, the mutations carried by patients vary due to the high heterogeneity of melanoma, which can lead to different responses to the existing targeted drugs among patients (36). Molecular characteristics of tumors can affect patients’ responses to treatment and even their survival, thus it is necessary to conduct additional investigation of the possible molecular mechanism of melanoma (37).

Previously, numerous investigations have revealed that lncRNAs are implicated in ferroptosis. LncRNA SLCO4A1-AS1 is highly expressed in pancreatic cancer. Knockout of SLCO4A1-AS1 can reduce the expression of SLC7A11 and increase sensitivity to the induction of ferroptosis (38). Cytoplasmic lncRNA p53 RRA influences the transcription of several metabolic genes and can promote ferroptosis through the activation of p53 (39). In lung cancer, lncRNA linc00336 has been shown to protect the tumor cells from ferroptosis by interacting with elavl1 to reduce the intracellular Fe^2+^ and lipid ROS levels (40). Thus, lncRNA is critical in the regulation of ferroptosis and might serve as a critical target for SKCM treatment and prognosis.

In this work, 16 DEfrlncRNAs were included in the prediction signature, of which several DEfrlncRNAs were reportedly related to the malignant progression and prognosis of various tumors, including linc00402 (41), linc02285 (42), linc00861 (43, 44), pik3cd-as1 (45), fam30a (46), linc01281 (47), and linc00996 (48). Overexpressed lncRNA pik3cd-as1 has been shown to promote LATS1 expression by competitively binding to miR-566, which resulted in the inhibition of hepatocellular carcinoma cell growth, invasion, and metastasis. The present study was the first to demonstrate that the 16 identified DEfrlncRNAs were related to the prognosis of melanoma, despite the underlying molecular mechanisms warrant further clarification.

The proposed classifier was used to accurately predict the SKCM patient survival more effectively compared to each clinicopathological risk factor. When these clinical features were used for stratification, the 16-DEfrlncRNAs classifier continued to be a robust prognostic model. As such, it provided prognostic value that served to complement the clinicopathological variables that were identified. Also, a novel nomogram was constructed for melanoma that utilized the 16 DEfrlncRNA RS as well as clinical features. This nomogram provided predictions that were more accurate for OS than the clinicopathological features that were identified.

To study the associations between tumor-infiltrating immune cells and the observed RS, we used the well-established CIBERSORT to evaluate tumor-infiltrating immune cells. We found that when the two risk groups were compared, the LR group exhibited a greater number of immune cells, such as CD8^+^ T cells, regulatory T cells (Tregs), M1 macrophages naive B cells, activated CD4^+^ memory T cells, and quiescent NK cells. These cells are indicators of a good prognosis. For example, melanomas with high infiltrated CD8^+^T cell content were reportedly associated with improved prognosis of patients (49, 50). In addition, elevated CD8^+^T cell infiltration could also predict prolonged OS in hepatocellular carcinoma (51). Upon the treatment with vaccines and ipilimumab, patients with melanoma harboring a pre-inflamed TME, like Treg infiltration, PD-L1, and IDO expression, might have a better long-term prognosis. Moreover, the infiltration of naïve B cells was shown to be associated with superior prognosis of patients with melanoma, lung adenocarcinoma, and neuroblastoma in several studies (52–54). As for activated CD4^+^ T memory cells, it is reported that their infiltration correlated with prolonged PFS and OS in melanoma patients (55). Lastly, a higher number of tumor-infiltrating NK cells was also reported to be associated with a good prognosis in metastatic melanoma (56). Moreover, due to the absence of infiltrated CD8^+^T in TME in the high RS group, the impaired induction of tumor cell ferroptosis might enable the survival of tumor cells, ultimately leading to tumor progression and worse survival of patients with melanoma. In the present study, we also discovered a correlation that existed between the RS and the immune checkpoint or IC50 sensitivity for different chemotherapeutic drugs. This information might help to employ differentiated therapeutic options between the two groups. For example, patients in the LR group exhibited higher immune checkpoint gene expression compared with those in the HR group, thus the former might be more sensitive to immunotherapy with PD-1/PD-L1 and CTLA-4 as its targets, and conventional chemotherapy drugs like nilotinib, methotrexate, rapamycin, and cisplatin might be more effective for the latter. To conclude, our findings indicated that DEfrlncRNAs might be implicated in anti-tumor immunity in melanoma.

However, our research had several limitations. First, conventional statistical methods were used to construct and evaluate 16-DEfrlncRNA risk prediction models associated with ferroptosis-related genes. Numerous investigations have demonstrated that these methods are feasible. Nevertheless, it is necessary to develop more advanced analytical technologies and methods to establish prognostic models with higher robustness and accuracy. Second, only data from the TCGA database were used to carry out the internal validation. In the future, external validation and validation utilizing data obtained from additional databases and clinical patients should also be conducted to assess the applicability of the predictive features more accurately. Finally, we just preliminarily explored the relationship between DEfrlncRNA and ferroptosis-associated genes. Thus, the precise underlying mechanisms of DEfrlncRNAs in SKCM, as well as the interactions with immune checkpoint and ferroptosis-related molecules, have not been completely elucidated. Therefore, additional investigation is needed to verify these observations.

## Conclusion

In conclusion, the DEfrlncRNA risk prediction model related to ferroptosis genes could independently predict the prognosis of melanoma patients with reasonable accuracy. These observations might help optimize the determination of therapeutic options in the treatment of melanoma. Further experimental verification is still needed to clarify the underlying associations between the observed molecular characteristics and functional significance.

## Data availability statement

The datasets presented in this study can be found in online repositories. The names of the repository/repositories and accession number(s) can be found below: https://portal.gdc.cancer.gov/, The Cancer Genome Atlas.

## Ethics statement

This study was reviewed and approved by Fourth Military Medical University. The patients/participants provided their written informed consent to participate in this study. Written informed consent was obtained from the individual(s), and minor(s)’ legal guardian/next of kin, for the publication of any potentially identifiable images or data included in this article.

## Author contributions

WG and ZL were in charge of the concept and design. XY provides administrative support. SG is in charge of data collection and assembly. SG and JC did the data analysis and interpretation. All authors contributed to the article and approved the submitted version.

## Funding

This work has received funding from National Natural Science Foundation of China (No. 81902791), Support Program of Young Talents in Shaanxi Province (No. 20200303) and Young Eagle Project of Fourth Military Medical University (No. 2019cyjhgwn).

## Conflict of interest

The authors declare that the review was finished in the absence of any commercial or financial relationships that could be construed as a potential conflict of interest.

## Publisher’s note

All claims expressed in this article are solely those of the authors and do not necessarily represent those of their affiliated organizations, or those of the publisher, the editors and the reviewers. Any product that may be evaluated in this article, or claim that may be made by its manufacturer, is not guaranteed or endorsed by the publisher.
