# Supplementary figures and images for: Identification and validation of ferroptosis-related lncRNA signature as a prognostic model for skin cutaneous melanoma
Source: Front Immunol. 2022 Sep 29;13:985051. doi: 10.3389/fimmu.2022.985051 (PMC9556814; doi:10.3389/fimmu.2022.985051)

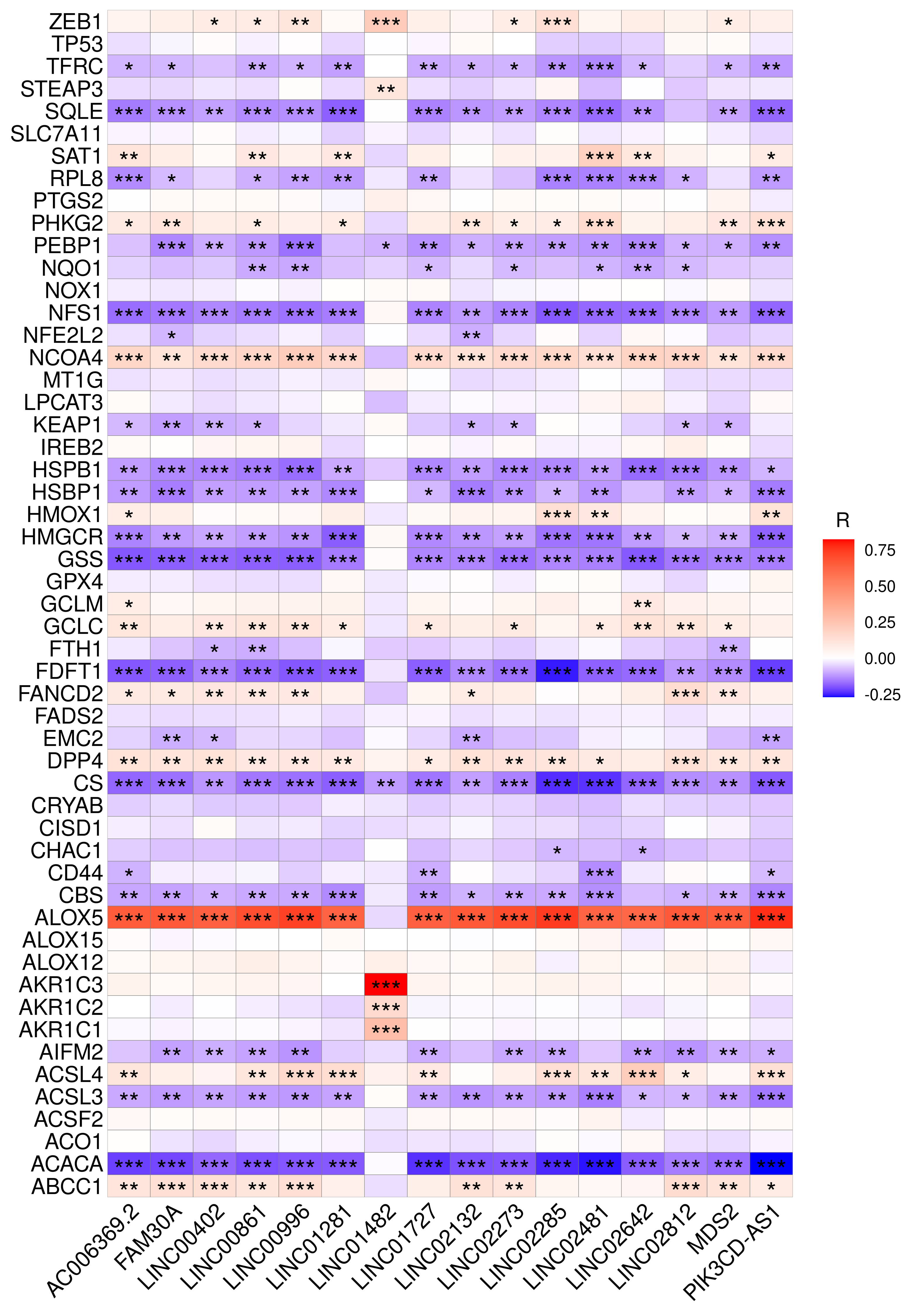

Supplement: Supplementary file 1 [file Image_1.tif]

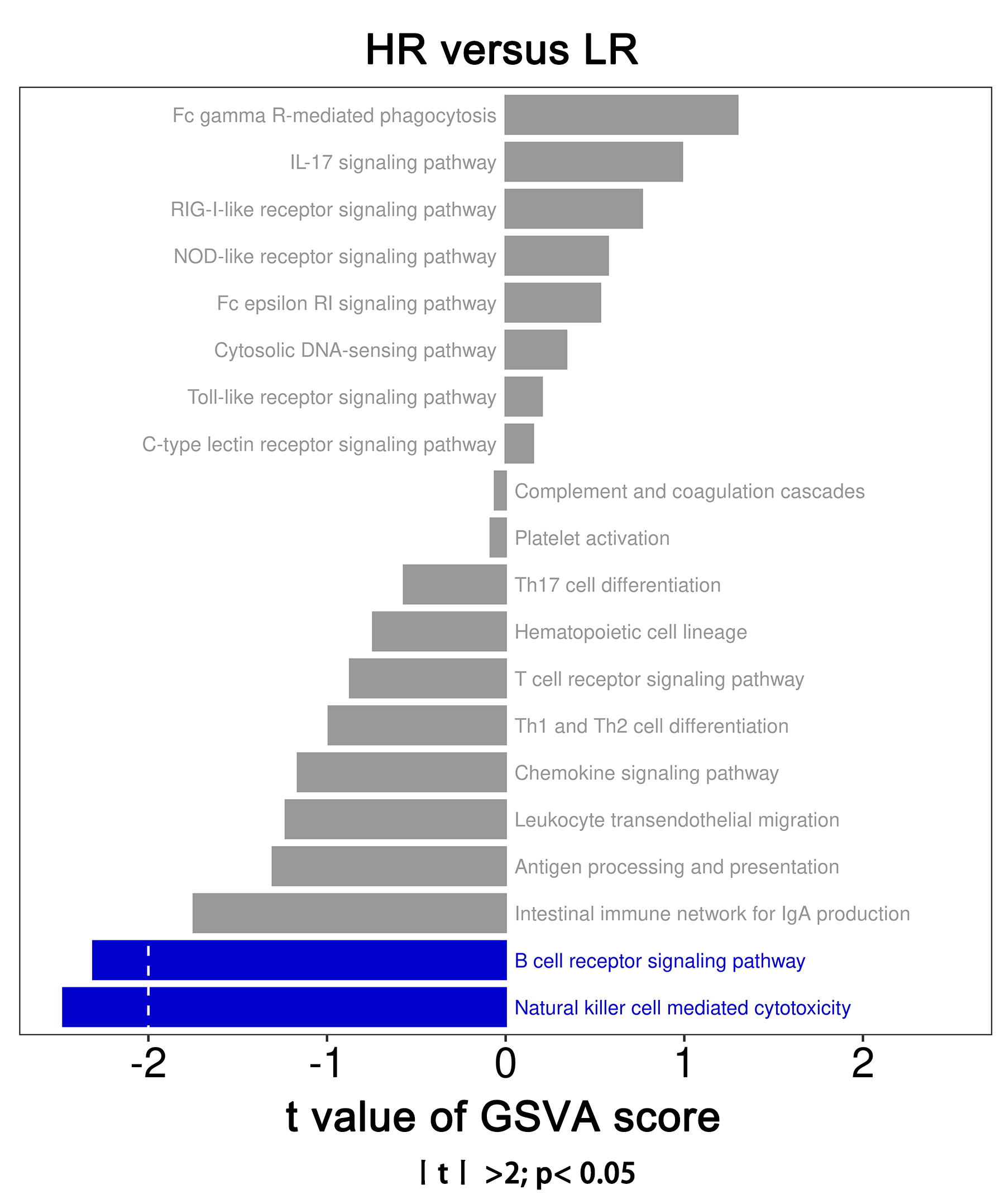

Supplement: Supplementary file 2 [file Image_2.tif]

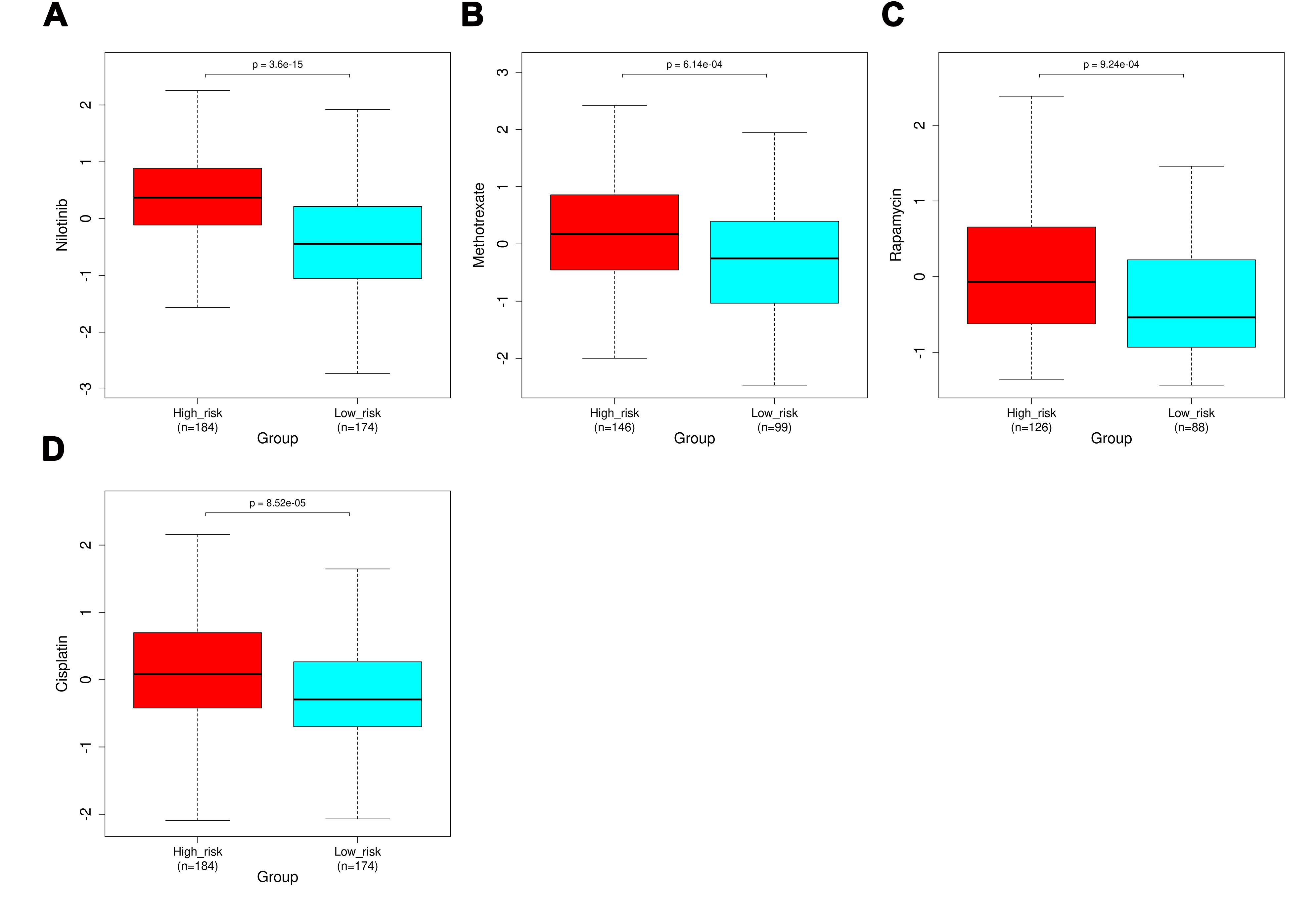

Supplement: Supplementary file 3 [file Image_3.tif]
