# Supplementary material for: Identification and validation of ferroptosis-related lncRNA signature as a prognostic model for skin cutaneous melanoma
Source: Front Immunol. 2022 Sep 29;13:985051. doi: 10.3389/fimmu.2022.985051 (PMC9556814; doi:10.3389/fimmu.2022.985051)
Supplement: Supplementary file 4 [file Table_1.docx]

Supplementary Table 1 A total of 98 frlncRNAs were identified positive correlation

| DElncRNA | fr-gene | R | p_value |
| --- | --- | --- | --- |
| SLC39A12-AS1 | AKR1C1 | 0.968883 | <0.01 |
| LINC00343 | AKR1C1 | 0.930892 | <0.01 |
| ERICH3-AS1 | AKR1C1 | 0.906733 | <0.01 |
| LINC02557 | AKR1C1 | 0.892743 | <0.01 |
| LINC01477 | AKR1C1 | 0.891395 | <0.01 |
| LINC00613 | AKR1C1 | 0.887826 | <0.01 |
| PTPRD-AS1 | AKR1C1 | 0.853518 | <0.01 |
| LINC00700 | AKR1C1 | 0.844015 | <0.01 |
| LINC02432 | AKR1C1 | 0.833118 | <0.01 |
| LINC01482 | AKR1C3 | 0.821001 | <0.01 |
| LINC02864 | AKR1C1 | 0.813831 | <0.01 |
| DANT1 | AKR1C1 | 0.798981 | <0.01 |
| PCED1B-AS1 | ALOX5 | 0.797585 | <0.01 |
| OTUD6B-AS1 | EMC2 | 0.793006 | <0.01 |
| FSIP2-AS2 | AKR1C1 | 0.786858 | <0.01 |
| LINC01901 | AKR1C1 | 0.776519 | <0.01 |
| PIK3CD-AS1 | ALOX5 | 0.772358 | <0.01 |
| LINC01801 | AKR1C1 | 0.771108 | <0.01 |
| LINC01574 | AKR1C1 | 0.767792 | <0.01 |
| EML4-AS1 | ALOX5 | 0.755502 | <0.01 |
| AC010969.1 | FADS2 | 0.749074 | <0.01 |
| LINC02285 | ALOX5 | 0.742034 | <0.01 |
| LINC02253 | AKR1C1 | 0.740651 | <0.01 |
| LINC01857 | ALOX5 | 0.728413 | <0.01 |
| LINC00996 | ALOX5 | 0.728079 | <0.01 |
| ADD3-AS1 | AKR1C3 | 0.72722 | <0.01 |
| LINC02077 | AKR1C1 | 0.721863 | <0.01 |
| AC069155.1 | MT1G | 0.714687 | <0.01 |
| CCR5AS | ALOX5 | 0.714127 | <0.01 |
| CEP250-AS1 | ALOX5 | 0.711874 | <0.01 |
| C9orf139 | ALOX5 | 0.707561 | <0.01 |
| ECE1-AS1 | ALOX5 | 0.703024 | <0.01 |
| LINC02876 | AKR1C1 | 0.702709 | <0.01 |
| LINC00528 | ALOX5 | 0.69995 | <0.01 |
| LINC02273 | ALOX5 | 0.698796 | <0.01 |
| LINC00861 | ALOX5 | 0.692179 | <0.01 |
| HYMAI | AKR1C1 | 0.691998 | <0.01 |
| LINC02089 | AKR1C1 | 0.691952 | <0.01 |
| AC244250.1 | PTGS2 | 0.681921 | <0.01 |
| LINC01624 | ALOX5 | 0.68192 | <0.01 |
| LINC01288 | AKR1C1 | 0.680668 | <0.01 |
| MIR223HG | ALOX5 | 0.675954 | <0.01 |
| AC023590.1 | ALOX5 | 0.675508 | <0.01 |
| ZNF22-AS1 | AKR1C1 | 0.671084 | <0.01 |
| AP003774.1 | ALOX5 | 0.669838 | <0.01 |
| LINC02560 | ALOX12 | 0.667052 | <0.01 |
| ITGB2-AS1 | ALOX5 | 0.665973 | <0.01 |
| DPP4-DT | DPP4 | 0.665339 | <0.01 |
| LINC02132 | ALOX5 | 0.664072 | <0.01 |
| LINC02812 | ALOX5 | 0.663073 | <0.01 |
| FAM30A | ALOX5 | 0.662926 | <0.01 |
| ADARB2-AS1 | AKR1C1 | 0.661328 | <0.01 |
| LINC01698 | FADS2 | 0.661246 | <0.01 |
| TRBV11-2 | ALOX5 | 0.661224 | <0.01 |
| LINC01414 | AKR1C1 | 0.660251 | <0.01 |
| LINC01215 | ALOX5 | 0.65683 | <0.01 |
| AC006369.2 | ALOX5 | 0.654582 | <0.01 |
| LINC02169 | FADS2 | 0.653492 | <0.01 |
| MDS2 | ALOX5 | 0.649483 | <0.01 |
| LINC01727 | ALOX5 | 0.641527 | <0.01 |
| LINC00402 | ALOX5 | 0.64123 | <0.01 |
| LINC01208 | FADS2 | 0.641165 | <0.01 |
| AC010105.1 | AKR1C1 | 0.640474 | <0.01 |
| RERE-AS1 | ALOX5 | 0.640207 | <0.01 |
| CARD11-AS1 | ALOX5 | 0.63823 | <0.01 |
| UBR5-AS1 | EMC2 | 0.636926 | <0.01 |
| LINC01281 | ALOX5 | 0.630221 | <0.01 |
| AC027319.1 | ALOX5 | 0.627808 | <0.01 |
| LINC02481 | ALOX5 | 0.626139 | <0.01 |
| ANKRD44-AS1 | ALOX5 | 0.621674 | <0.01 |
| LINC01825 | ALOX5 | 0.621659 | <0.01 |
| TMEM212-AS1 | HMOX1 | 0.618424 | <0.01 |
| LINC02642 | ALOX5 | 0.617926 | <0.01 |
| LASTR | STEAP3 | 0.617774 | <0.01 |
| LINC02382 | AKR1C1 | 0.616829 | <0.01 |
| PLUT | ACSF2 | 0.615109 | <0.01 |
| LINC02573 | AKR1C1 | 0.614452 | <0.01 |
| LINC02821 | ALOX15 | 0.614143 | <0.01 |
| MMP2-AS1 | ALOX5 | 0.612759 | <0.01 |
| COPDA1 | ALOX5 | 0.612238 | <0.01 |
| LINC01094 | ALOX5 | 0.612093 | <0.01 |
| PWRN1 | AKR1C1 | 0.610332 | <0.01 |
| LINC02241 | AKR1C1 | 0.610248 | <0.01 |
| MIR181A1HG | AKR1C1 | 0.6093 | <0.01 |
| MIR4432HG | ALOX5 | 0.608956 | <0.01 |
| LINGO1-AS2 | FADS2 | 0.6089 | <0.01 |
| LINC02830 | AKR1C1 | 0.607406 | <0.01 |
| TNK2-AS1 | ALOX5 | 0.605964 | <0.01 |
| RBM38-AS1 | ALOX5 | 0.605949 | <0.01 |
| LINC02413 | ALOX5 | 0.605411 | <0.01 |
| LINC01891 | ALOX5 | 0.604932 | <0.01 |
| LINC02648 | ALOX5 | 0.603698 | <0.01 |
| SNHG6 | RPL8 | 0.603571 | <0.01 |
| FAM225B | ZEB1 | 0.602851 | <0.01 |
| LINC01754 | ALOX5 | 0.602195 | <0.01 |
| LINC02325 | ALOX5 | 0.601385 | <0.01 |
| LINC02295 | ALOX5 | 0.600814 | <0.01 |
| LINC00885 | FADS2 | 0.600229 | <0.01 |
